# The tin content of lead inclusions in ancient tin-bronze artifacts: a time-dependent process?

**DOI:** 10.1107/S1600576724002218

**Published:** 2024-05-10

**Authors:** Gilberto Artioli, Alessandro Fontanari, Ivana Angelini, Chiara Lucarelli, Martin Etter, Henrik S. Jeppesen, Sana Shilstein, Sariel Shalev

**Affiliations:** a Department of Geosciences, Università di Padova, Via Gradenigo 6, Padova 35131, Italy; b Department of Cultural Heritage, Università di Padova, Piazza Capitaniato 7, Padova 35139, Italy; c FS-PETRA-D, Deutsche Elektronen-Synchrotron DESY, Notkestrasse 85, Hamburg 22607, Germany; d Weizmann Institute of Science, Rehovot, Israel; Australian Nuclear Science and Technology Organisation, Lucas Heights, Australia

**Keywords:** Sn–Pb alloys, ancient Sn–Cu bronzes, authentication, cultural heritage, X-ray diffraction

## Abstract

In antiquity, Pb was a common element added in the production of large bronze artifacts, especially large statues, to impart fluidity to the casting process. As Pb does not form a solid solution with pure Cu or with the Sn–Cu alloy phases, it is normally observed in the metal matrix as globular droplets embedded within or in interstitial positions among the crystals of Sn-bronze (normally the α phase) as the last crystallizing phase during the cooling process of the Cu–Sn–Pb ternary melt. The disequilibrium Sn content of the Pb droplets has recently been suggested as a viable parameter to detect modern materials [Shilstein, Berner, Feldman, Shalev & Rosenberg (2019). *STAR Sci. Tech. Archaeol. Res.* **5**, 29–35]. The application assumes a time-dependent process, with a timescale of hundreds of years, estimated on the basis of the diffusion coefficient of Sn in Pb over a length of a few micrometres [Oberschmidt, Kim & Gupta (1982). *J. Appl. Phys.* **53**, 5672–5677]. Therefore, Pb inclusions in recent Sn-bronze artifacts are actually a metastable solid solution of Pb–Sn containing ∼3% atomic Sn. In contrast, in ancient artifacts, unmixing processes and diffusion of Sn from the micro- and nano-inclusions of Pb to the matrix occur, resulting in the Pb inclusions containing a substantially lower or negligible amount of Sn. The Sn content in the Pb inclusions relies on accurate measurement of the lattice parameter of the phase in the Pb–Sn solid solution, since for low Sn values it closely follows Vegard’s law. Here, several new measurements on modern and ancient samples are presented and discussed in order to verify the applicability of the method to the detection of modern artwork pretending to be ancient.

## Abstract 2

The disequilibrium Sn content in Pb inclusions present in ancient (Sn,Cu)-bronze samples may in principle be used for authentication purposes. The present article explores the applicability of a method based on precise diffraction measurement of the Pb lattice parameter.

## 1. Introduction

**Figure 1:**
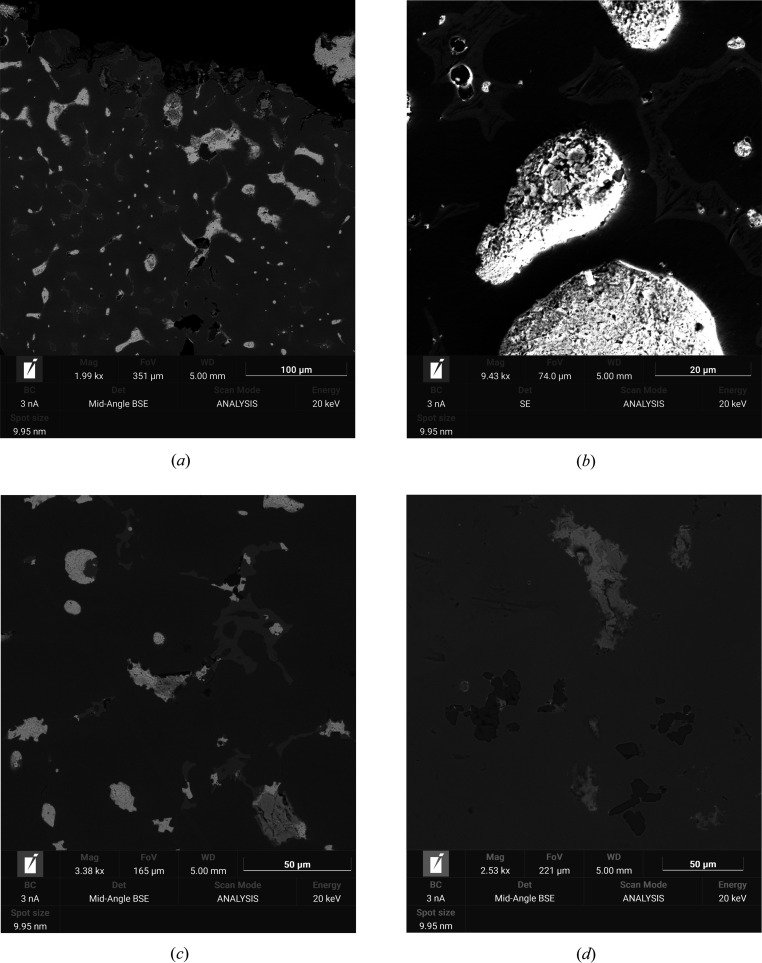
Backscattered and secondary electron images of Pb droplets and inclusions in modern and ancient bronzes. Sample labels correspond to those in Table 3 ▸: (*a*) sample S3-BronzeMg1 showing white Pb droplets, pale grey interstitial δ phase with Sn content around 31–34 wt% and a dark grey matrix formed by the α phase with Sn content around 6–12 wt%; (*b*) sample S3-BronzeMgB2 showing the dark α phase neatly separating the Pb-rich droplets from the Cu–Sn bronze δ phase; (*c*) sample S3-Bronze39, which is a Cu–Sn–Pb–Zn alloy, with the dark matrix α phase having a composition around Sn 4–6 wt% and Zn 15–18 wt%, and the light grey δ phase having a composition close to Sn 25–28 wt% and Zn 4–6 wt%, where the small black segregations are Cu sulfides; and (*d*) sample S3-Bronze57, which is also a Cu–Sn–Pb–Zn alloy, with the matrix α phase having a composition around Zn 8–9 wt% and Sn 4–6 wt%, where the dark grey inclusions are all Zn sulfides.

(Cu,Sn)-based metal alloys in large bronze artifacts such as statues or architectural components may contain other elements added to modify the physical properties, such as Pb or Zn, or minor impurities derived from the unrefined metal sources. In Roman times, Pb was added to ancient bronze statues in substantial quantities (5–20 wt%) to reduce the melting temperature and the viscosity of the molten metal, thus facilitating casting operations (Scott *et al.*, 1994 ▸; Scott, 2002 ▸; Thomas, 2007 ▸; Ruusila *et al.*, 2013 ▸). The presence of Pb does not affect the microstructure during solidification, as it is virtually immiscible in the solid Cu–Sn phases. Pb is therefore invariably confined in residual globules and droplets crystallizing at low temperature (*T* < 326°C) (Fig. 1 ▸). They may be of variable size and well distributed in low-Pb bronzes, or large and irregular in high-Pb bronzes, depending mostly on the homogeneity and the cooling rate (Bernabale *et al.*, 2019 ▸).

The globules of Pb, if present in large quantities, may form structural discontinuities of the hard Cu–Sn matrix, even leading to mechanical failure. Therefore, ternary alloys of Pb–Sn–Cu are not common in artifacts needing high strength, such as blades (swords, knives). Rather, ternary alloys are and were commonly used for symbolic artifacts like large statues, or objects needing substantial ductility during working, such as laminae or metal cists.

The low-melting phase is generally found to be composed of pure Pb (melting point = 327°C), whose crystal shape, size and distribution largely depend on the cooling rate (Nyyssönen *et al.*, 2012 ▸; Ruusila *et al.*, 2013 ▸). At low cooling rates (*e.g.* sand casting, pouring in bivalve moulds) there is enough time for the growth and agglomeration of the Sn-bronze dendrites, which form large-sized microstructures. Pb as the last crystallizing phase is confined at the grain interstices. In the case of fast cooling (*e.g.* quenching in oil or water), freezing is so fast that the Pb is trapped as fine droplets dispersed throughout the solid matrix of the α, β or δ phases of the Cu–Sn alloy.

Since during the cooling process the melt is rich in Sn and Pb, a binary alloy of Pb–Sn could in principle solidify with the Sn solubility in Pb reaching ∼3 at.% at ambient temperature. This aspect has never been adequately considered in the literature. However, recently Shilstein *et al.* (2019 ▸) confirmed experimentally the presence of an appreciable content of Sn in the Pb inclusions of quenched bronzes, eventually reaching thermodynamic equilibrium following unknown kinetics. It was proposed that the Sn content in Pb segregations could potentially be used as an authentication method for spotting modern copies or forgeries of ancient bronzes. However, because of the small size of the Pb droplets, it is virtually impossible to separate them by mechanical or chemical separation for adequate compositional measurements. Even microbeam analyses are often biased by matrix effects and prove unreliable (Armstrong, 1991 ▸). In energy-dispersive spectro­scopy (EDS), for example, the effect of matrix absorption depends on the distance X-rays must travel from the generating point until they exit the sample. Therefore, the composition of microparticles estimated by the microprobe is often biased by the surrounding environment. The possibility of reliably assessing the Sn content in the Pb inclusions thus relies on the accurate measurement of the lattice parameter of the α phase in the Pb–Sn solid solution, since for low Sn values it should closely follow Vegard’s law (Fecht & Perepezko, 1989 ▸).

The aim of this work is to verify the applicability of the method proposed by Shilstein *et al.* (2019 ▸), highlighting its potential and limitations. The samples taken into consideration include several ancient Pb-containing bronzes of classical and medieval age, modern Pb–bronze alloys, and artificially produced solid solutions of selected Pb–Sn compositions, especially selected for calibration of the lattice-parameter–composition relations and validation of the linearity of Vegard’s law in the low-Sn compositional range of interest. The amount of Sn in the Pb samples is determined by measuring the α-phase cell parameter by laboratory and synchrotron X-ray powder diffraction (XRPD) measurements.

## 2. Verification of Vegard’s law in the f.c.c. α phase of Pb–Sn composition, in the low-Sn regime

In a random substitutional solid solution of elements with the same crystal structure, a linear relation between the lattice parameter and concentrations, known as Vegard’s law, is generally observed (Denton & Ashcroft, 1991 ▸; Magomedov, 2020 ▸). For a binary alloy *A*–*B*, the lattice parameter could be described by the following equation (Sidot *et al.*, 2005 ▸):

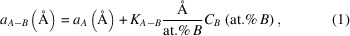

where *a*_*A*−*B*_ is the lattice parameter of the alloy, *a*_*A*_ is the lattice parameter of the pure metal *A*, *C*_*B*_ is the atomic fraction of the solute *B* and *K*_*A*−*B*_ is the constant of proportionality characteristic for the *A*–*B* alloy.

This means that, in the first approximation, the lattice parameter of the alloy is controlled by the relative size of the exchanged atoms. As a rule of thumb, this is not strictly applicable to every alloy, but in the case of Pb–Sn binary alloys its validity has been demonstrated up to 5 at.% of Sn substituted in a Pb lattice (Tyzack & Raynor, 1954 ▸). The solubility limit at ambient temperature of Sn in Pb is stated as not exceeding 3 at.%. Going beyond this value is only possible by annealing and subsequent quenching. However, above 5 at.% there is a breakdown of the solid solution and accurate lattice spacings could not be obtained (Tyzack & Raynor, 1954 ▸). Regarding the ternary alloy Cu–Sn–Pb or the quaternary alloy Cu–Sn–Pb–(Zn), the solubility of Cu and Zn in Pb is extremely low at room temperature (Sidot *et al.*, 2005 ▸). Pb and Sn also have a low affinity for the Cu–Zn alloy phases (brass).

To verify and confirm the validity of Vegard’s law for the binary alloy Pb–Sn, a series of alloy samples with different compositions were prepared, and the lattice parameter of Pb was measured using XRPD.

## 3. Materials and methods

### 3.1. Materials

The alloys and pure metals considered in this study are listed in Tables 1–3.

The first set of samples (Table 1 ▸) refers to artificial binary Pb–Sn alloys used for the validation of Vegard’s law for Pb–Sn solid solution in the low-Sn region. The Sn composition ranges from 1.23 to 63 at.% (0.72–50 wt%). Apart from the 50 wt% Pb–Sn alloy, which is a commercial soldering alloy, these samples were produced in vacuum-sealed Pyrex tubes (*p* < 10^−3^ mbar) from Sn and Pb with a purity close to 99.99 wt% (Nova Elements s.a.s.). The metals were melted in a temperature-controlled furnace (350°C) and immediately quenched in cold water. Shattering of the Pyrex tube was observed on quenching.

The second set of samples (Table 2 ▸) refers to artificial ternary Cu–Sn–Pb alloys, prepared as modern replicas of leaded Sn-bronze (Pb-bronze), used for comparison with historical bronze samples. The modern Pb-bronze alloy composition has a fixed Cu:Sn ratio at 90:10 wt% and a variable Pb content in the range 4.9–16 wt%, to investigate a possible dilution effect of Pb in the bronze matrix. The alloy composition was chosen in accordance with the average Roman bronze composition (Dungworth, 1997 ▸), containing 8–10 wt% of Sn and variable Pb content ranging from 5 to 15 wt%. The casting of the ternary alloys took place in a sealed graphite crucible at a temperature of 950°C, to avoid oxidation. The melt was poured into a graphite mould and immediately quenched in cold water. The final samples of molten Pb-bronze are spherical droplets with an average diameter of 5 mm.

The third set of samples (Table 3 ▸) refers to historical samples covering a wide time span, from the Hellenistic period (∼300 BC) to the early 20th century (1900 AD).

### 3.2. Methods

#### 3.2.1. Laboratory X-ray diffraction (L-XRD)

The study of lattice parameters of the alloys was performed by XRD with a Philips X’Pert PRO instrument equipped with an X’Celerator detector and a Bragg–Brentano HD monochromator from Panalytical. The diffractometer worked in reflection mode (Bragg–Brentano geometry), using a Co source [λ(*K*α_1_) = 1.78901 Å] and working conditions of 40 kV and 40 mA. A step scan of 0.017° was adopted, with 100 s step^−1^ counting time, a scan angle of 2θ = 3–85° and a spinning sample. The lattice parameter of α-lead was calculated using the Le Bail method (Le Bail, 2005 ▸), which involved fitting the entire XRD pattern using the *Profex–BGMN* software (Doebelin & Klee­berg, 2015 ▸). The Le Bail method was chosen for extract­ing the lattice parameter to achieve the highest possible precision, as it can effectively describe textured phases and provide the best fit possible for the peak profile (Peterson, 2005 ▸). Additionally, quantitative determination of each metal­lic phase was carried out using a Rietveld-type refinement approach (Rietveld, 1969 ▸). The surfaces of the metal samples analysed were gently manually polished before measurement with fine-grain sandpaper (1200 grit) using ethanol. The analysed surface area of the sample used for the measurement was 5 × 5 mm. To assess the theoretical accuracy achievable with this experimental configuration, a reference standard of LaB_6_ (NIST SRM 660b) was measured using the same settings. The difference between reference and measured values is of the order of 5 × 10^−4^ Å. The estimated standard uncertainty is reduced by repeated measurements of the same re-mounted sample to average out the sample-displacement contribution.

#### 3.2.2. Synchrotron X-ray diffraction (S-XRD)

Several samples prepared for this investigation were also measured at the Powder Diffraction and Total Scattering Beamline P02.1 of PETRA III, DESY, Hamburg, Germany, using the Debye geo­metry, with the samples enclosed in glass capillaries, and a wavelength of 0.207316 Å (Dippel *et al.*, 2015 ▸). The sample-to-detector distance was set to a large value (2190 mm) in order to obtain the highest possible angular resolution. The samples consisted of metal fragments scraped from the surface of the polished metal used for the L-XRD experiments. They were inserted in glass capillaries with variable diameter in the range 0.5–1 mm. The reference standard of LaB_6_ (NIST SRM 660b) was used to calibrate the wavelength and the sample–detector distance. Rietveld refinement and Le Bail fitting of the S-XRD measured patterns were performed using the *Profex–BGMN* software and adopting the fundamental parameter approach (Doebelin & Kleeberg, 2015 ▸).

## 4. Results

### 4.1. Binary alloys of Pb–Sn (sample set 1)

The lattice parameters of pure Pb and of the binary Pb–Sn alloys of sample set 1 resulting from the described whole pattern fitting procedures are listed in Table 4 ▸. Calibration of pure Pb (0 at.% Sn) was performed by repeated measurements and analyses of pure Pb samples. The resulting lattice parameter [*a*_Pb_ = 4.9522 (1) Å] is slightly larger than the commonly reported value for Pb at 25°C [*a*_Pb_ = 4.94006 (2) Å, Straumanis (1949 ▸); *a*_Pb_ = 4.9506 (1) Å, Swanson & Tatge (1953 ▸); see also JCPDS card 04-0686; *a*_Pb_ = 4.9511(1) Å, Shilstein *et al.* (2019 ▸)]. A better consistency in the measured lattice parameter of Pb with the commonly reported value is achieved through S-XRD, as shown in the comparison presented in Table 6.

The small standard deviation of the repeated measurements of pure Pb suggests a high precision for the method employed, but with a relatively low accuracy, with the considerable difference of the lattice-parameter value possibly caused by systematic discrepancies in the annealing and thus in the microstructure of the samples. This is why textured metals are avoided for line and shape calibration in powder diffraction.

**Figure 2:**
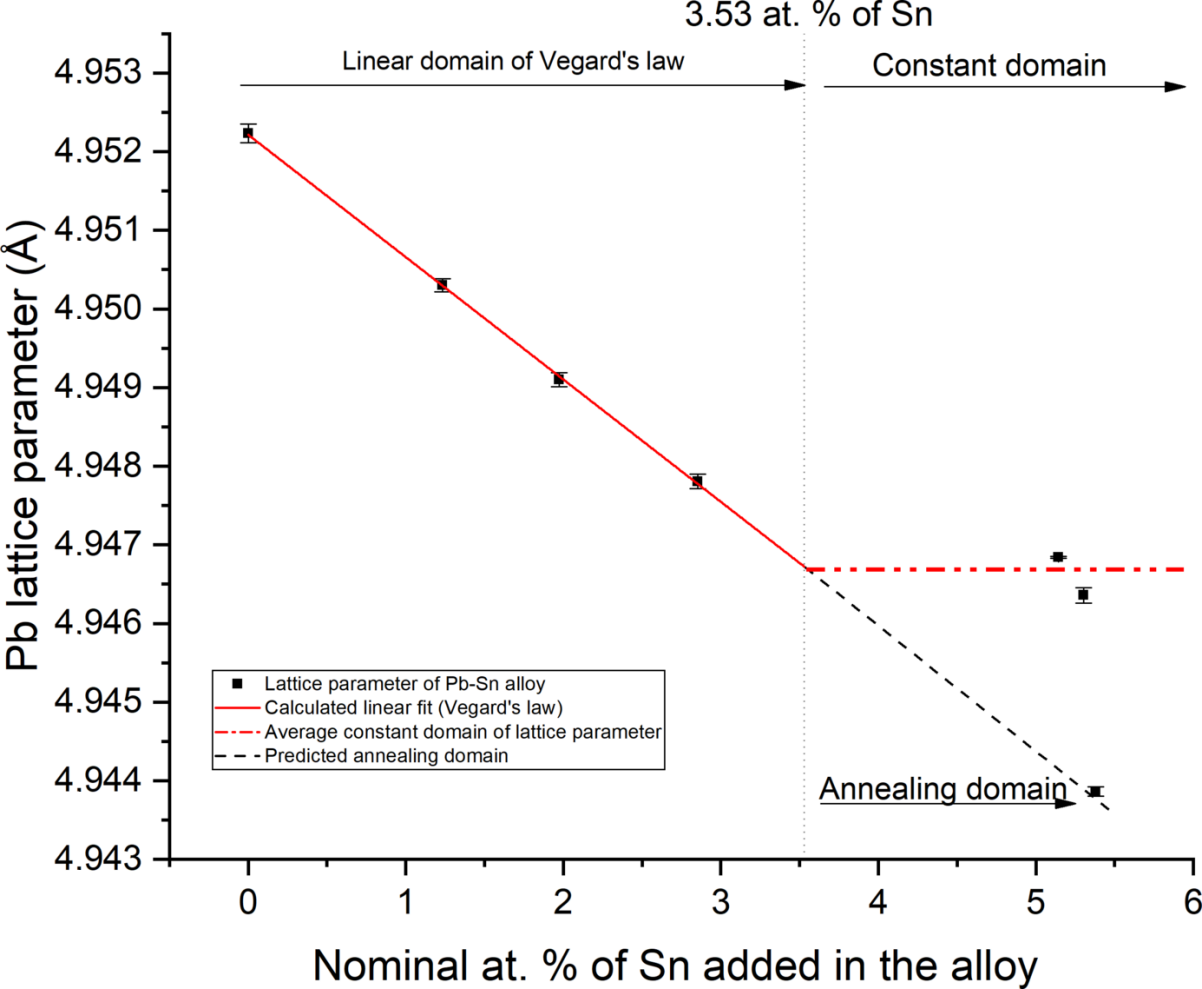
Vegard’s law calculated from the experimental lattice parameter data in Table 4 ▸.

A linear relation is found for the four Pb–Sn alloy samples in the range 0–2.85 at.% of added Sn (Fig. 2 ▸). For higher quantities of added Sn, in the range 5.14–63.11 at.%, the measured lattice parameter is virtually constant over the whole compositional range, with an average value of *a*_PbSn_ = 4.9467 (2) Å. The linear regression of the first four samples of Table 4 ▸ leads to the following equation, confirming the validity of Vegard’s law in the low-Sn region:

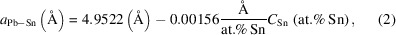

with regression coefficient *R* = 0.9996. There is a slight difference between the result of this work and the slope and intercept (*i.e.* the lattice parameter of pure Pb) of previous work (Tyzack & Raynor, 1954 ▸).

The intercept of the two experimental lines (Fig. 2 ▸) leads to a solubility limit of Sn in Pb of 3.53 at.%, in agreement with previous work. The results indicate that, in Pb–Sn alloys containing more than 3.53 at.% of Sn, excess Sn is essentially present as a pure metal and the diffraction peaks of β-tin can be observed. Phase quantification of Sn and Pb from the diffraction patterns was performed with Rietveld refinements, and the results are listed in Table 4 ▸.

The only way to overcome the observed solubility limit of Sn in Pb is through a prolonged heat treatment (annealing) followed by a rapid quenching. In fact, following this procedure it is possible to reach a content of Sn in Pb of up to 5 at.% (Tyzack & Raynor, 1954 ▸), which is considered thermodynamically metastable in equilibrium conditions. To confirm this possibility, the sample S1-PbSn63 with 63.11 at.% Sn was heat treated at 90°C for 6 h and immediately quenched in cold water. The measured cell parameter of the annealed sample (S1-PbSn63_an) is *a*_PbSn_annealed_ = 4.9439 (1) Å, and by using the previously determined relationship [equation (2)] an Sn content in Pb of 5.32 at.% was obtained. This is possibly the maximum Sn content in Pb obtainable through fast cooling in the laboratory.

### 4.2. Lead inclusion in ternary alloy Cu–Sn–Pb (sample set 2)

**Figure 3:**
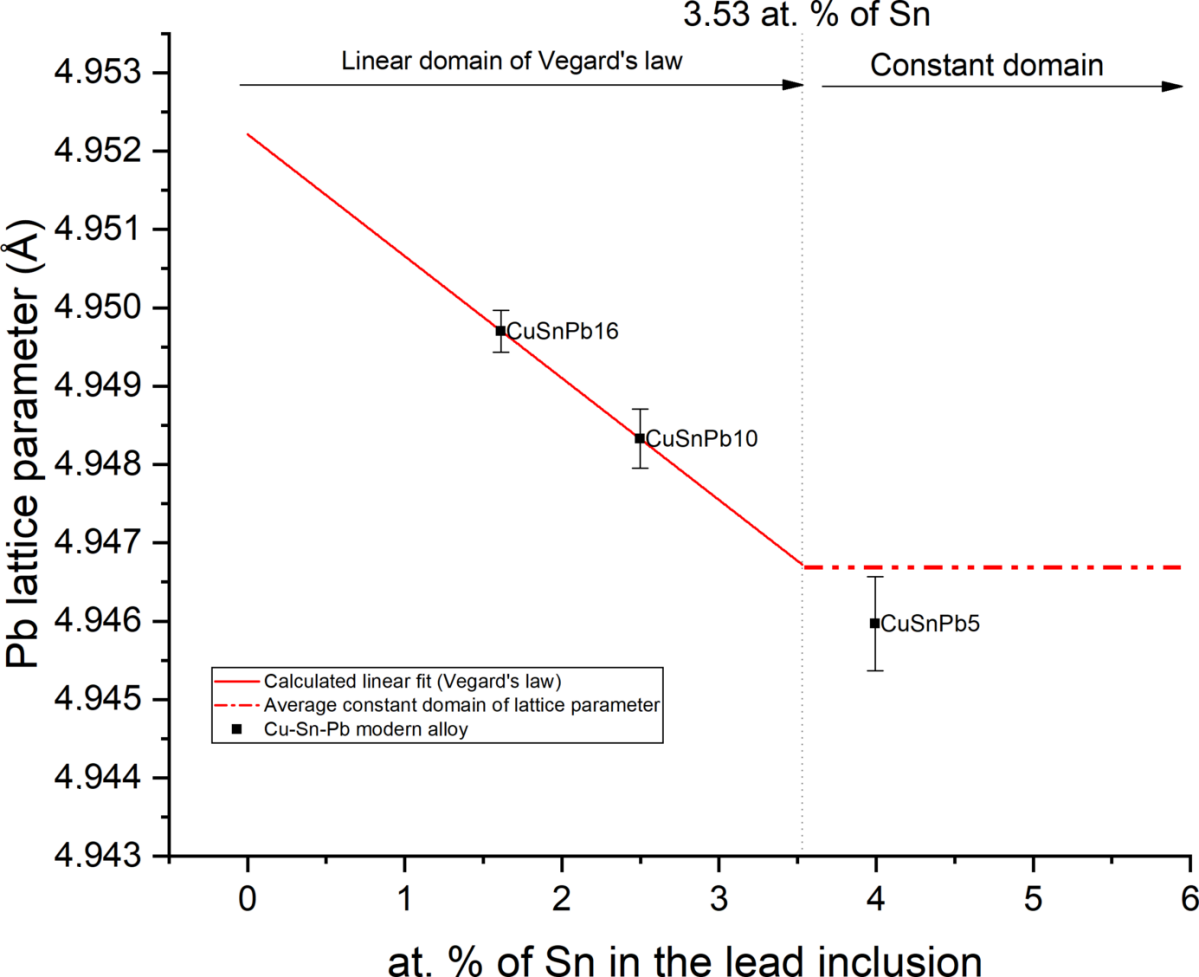
Extrapolated values of the atomic percentage of Sn from Pb lattice parameters of modern ternary bronze alloy Cu–Sn–Pb.

The lattice parameters of Pb in the modern ternary alloys of Cu–Sn–Pb (sample set 2) are reported in Table 5 ▸. The cell parameters measured for the Pb segregations do not correspond to pure Pb. Assuming that the Pb inclusions of the ternary Cu–Sn–Pb alloy are formed by Sn–Pb solid solutions, it is possible to calculate the Sn content by using the previously obtained calibration [equation (2)]. The values are graphically reported in Fig. 3 ▸.

The Sn content in the Sn–Pb inclusions is inversely proportional to the Pb amount added to the Sn-bronze. This indicates a dilution effect proportional to the Pb content. A high Pb content in the bronze (>16 wt% Pb) forms inclusions with a limited amount of Sn (1.6 at.% Sn), whereas a low Pb content in the bronze (<5 wt% Pb) forms inclusions ap­proach­ing the observed solubility limit of Sn in Pb (3.5 at.% Sn).

### 4.3. Historical samples (sample set 3)

Several historical Sn-bronze and Sn-brass samples were selected (Table 6 ▸) to investigate the time dependency of the Sn content in Pb inclusions, as proposed by Shilstein *et al.* (2019 ▸).

The lattice parameter of Pb in the ternary alloy Cu–Sn–Pb was determined, both with a conventional laboratory X-ray source (L-XRD) and with a synchrotron light source (S-XRD). The pure Pb sample and Pb–Sn alloy with a high Sn content were also measured with both experimental conditions for comparison. The two sets of measurements are substantially in agreement, despite a small systematic shift in the absolute values of the resulting lattice parameters. By reconsidering the value of the intercept and by keeping the slope of the fit unchanged, it is possible to estimate the Sn content in Pb inclusions (Table 6 ▸). The following equation was used:

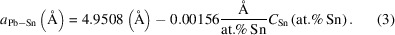

The estimated Sn content of Pb inclusions in all the historical samples is on average less than 0.5 at.%, which is close to or less than the estimated relative error. The results confirm that the Pb inclusions in historical ternary and quaternary alloys made of Cu–Sn–Pb–(Zn) are composed of very low Sn or pure Pb, independent of the time of casting.

## 5. Discussion and conclusions

The present study confirms the linearity of the relationship between the lattice parameter of Pb–Sn binary alloys present as inclusions in Sn-bronze and the atomic concentration of Sn in the alloy, with a solubility limit of Sn in Pb close to 3.5 at.%. The reported calibrated relation can be used practically to estimate the Sn content in Pb inclusions in Sn-bronze artifacts made of ternary alloy Cu–Sn–Pb.

In modern Sn-bronze cast with variable Pb content, Sn is predominantly present in Pb inclusions and segregations. A dilution effect is observed in high-Pb alloys, with the average Sn content in the Pb inclusions being substantially lower than the measured solubility limit of Sn in Pb. It is likely that varying the Sn content in bronze at constant Pb contents could have a similar effect of dilution.

In ancient Sn-bronze artifacts, Sn in Pb droplets is present in amounts close to the uncertainty of the measurement (Sn ≤ 0.5 at.%), so that the Pb inclusions contain at most a very low Sn content. All measured historical samples show this feature, independent of the alleged time of manufacturing. Even recent samples containing Zn, and therefore composed of quaternary Cu–Sn–Pb–(Zn) alloys (samples S3-Bronze39 and S3-Bronze57), seem to behave similarly to ternary Cu–Sn–Pb alloys, possibly because of the even lower solubility of Zn in Sn–Pb solid solutions. The overall conclusions can thus be extrapolated to brass.

Concerning the potential applicability of the method proposed by Shilstein *et al.* (2019 ▸) for the identification of recent Sn-bronze artifacts containing Pb, the cooling rate should be taken into consideration, since it may strongly affect the Sn content in the Pb inclusions. The present work indicates that an Sn content in Pb inclusions that is close to the solubility limit (Sn = 3.5 at.%) is experimentally confirmed only for rapidly quenched samples, where the Pb inclusions are very small (*d* < 2 µm). At low cooling rates, the Pb inclusions may be substantially larger (*d* > 50 µm, see Fig. 1 ▸), and this may influence the Sn diffusion. Metal alloys reheated in the presence of excess Sn and quenched (*i.e.* sample S1-PbSn63_an) maintain quantities of Sn close to or above the solubility limit. However, this is probably hard to achieve for old Sn-bronzes, which commonly do not contain excess Sn, as below 520°C all Sn is virtually included in the α and δ phases of the bronze matrix.

As a matter of fact, to date, the only measured ancient sample containing quantities of Sn close to the solubility limit in Pb inclusions is an ancient bronze coin [sample 14 of Shilstein *et al.* (2019 ▸)], which is a small object that could potentially be originally quenched, or recently reheated and quenched. All studied ancient samples from large objects such as bronze statues and architectural elements, including those investigated in the present work, proved to contain very low Sn or pure Pb inclusions within the sensitivity of the XRD measurements. This is probably related to the slow cooling process that commonly follows the casting operations of large artifacts, combined with the time-dependent kinetics of unmixing and diffusion of Sn from the micro- and nano-inclusions of Pb to the matrix. The mechanism must be similar to that reported for the relatively fast decay in the mechanical properties of Pb–Sn solders with time, for which it was proposed that the microstructure of the alloy changes with room-temperature ageing (Dreyer & Müller, 2000 ▸), possibly due to the precipitation of Sn within the Pb dendrites as a consequence of the decrease in solubility of Sn in Pb from ∼18 wt% at 183°C to 3 wt% below 50°C (Callister, 1997 ▸). At least three driving forces are proposed to act towards the microstructural changes, commonly described as coarsening (Harris *et al.*, 1991 ▸): the classical diffusion mechanism described by Ficks’ law, the surface tension of the inclusions and the thermo-mechanical stresses developed by different thermal expansions of the involved phases (Dreyer & Müller, 2000 ▸). The granular microstructure observed in the largest Pb inclusions in bronzes [Fig. 1 ▸(*b*)] is indeed consistent with such mechanisms. The thermal diffusion coefficients of Sn in Pb and Pb in Sn, however, are very low at room temperature; therefore, it is not straightforward to justify the rapid degradation of Pb–Sn soldering (reported within tens of years), or the rapid change in composition of the Pb inclusions in bronzes, assessed over the order of a few hundred years (Shilstein *et al.*, 2019 ▸; Oberschmidt *et al.*, 1982 ▸).

Another possible limitation to the authentication of large bronze artifacts derives from the difficulties in conducting bulk measurements with X-rays. Typically, samples are taken from the surface, which could lead to biased results due to surface alteration products. Finally, caution should be taken when extracting the lattice parameters of the Pb inclusions from powder diffraction when litharge or the δ phase of Sn-bronze are present, because peak overlap may cause serious biases in the results.

## Figures and Tables

**Table 1:** Pb–Sn artificially produced binary alloy samples for the validation of the linearity of Vegard’s law (sample set 1)

Sample name	Sn added (wt%)	Sn added (at.%)	Note
S1-PbSn0	0	0	Pure lead
S1-PbSn1	0.72	1.23	–
S1-PbSn2	1.16	1.97	–
S1-PbSn3	1.69	2.85	–
S1-PbSn5	3.00	5.14	–
S1-PbSn5b	3.17	5.30	–
S1-PbSn8	4.76	8.02	–
S1-PbSn9	5.47	9.02	–
S1-PbSn37	26.10	37.70	–
S1-PbSn63	50.00	63.11	Commercial solder alloy

**Table 2:** Cu–Sn–Pb artificially produced modern ternary alloys (sample set 2)

Sample name	Pb added (wt%)	Cu added (wt%)	Sn added (wt%)
S2-CuSnPb0	0.00	90.00	10.00
S2-CuSnPb5	4.90	85.59	9.51
S2-CuSnPb10	9.38	81.56	9.06
S2-CuSnPb16	16.04	75.56	8.40

**Table 3:** Historical Cu–Sn–Pb and Cu–Sn–Pb–Zn alloys (sample set 3) The mean composition was measured by scanning electron microscopy (SEM)–EDS area analysis. The term N.d. means not detected (detection limit of Zn < 0.1 wt%).

Sample name	Period	Type, origin	Pb measured (wt%)	Cu measured (wt%)	Sn measured (wt%)	Zn measured (wt%)
S3-Bronze39	1901 AD	Fountain of the Naiads, Rome	5.78	70.46	3.57	19.27
S3-Bronze57	1912 AD	Monument of General Cialdini, Castelfidardo	2.92	80.11	5.60	8.27
S3-Bronze98	Medieval period	St Mark Lion, Venice	4.84	87.74	6.70	0.62
S3-Bronze99	Possible modern restoration	St Mark Lion, Venice	2.91	87.16	7.05	2.52
S3-BronzeMg1	Hellenistic period	Bronze statue, Tuscany	7.17	79.15	11.47	N.d.
S3-BronzeMg5	Hellenistic period	Bronze statue, Tuscany	17.08	74.24	9.29	N.d.
S3-BronzeMgB2	Hellenistic period	Bronze statue, Tuscany	17.24	63.38	13.37	N.d.
S3-BronzeMgB4	Hellenistic period	Bronze statue, Tuscany	21.99	71.24	9.86	N.d.

**Table 4:** Measured lattice parameters of samples of set 1, with phase quantities as resulting from the Rietveld-type refinements The term N.d. means not detected (detection limit of Sn < 0.1 wt%).

			XRD phase quantity	
Sample name	Sn content added in the alloy (at.%)	Lattice parameter of Pb measured from XRD (Å) (e.s.d. = 10^−4^)	Sn (at.%)	Pb (at.%)
S1-PbSn0	0	4.9522	N.d.	100
S1-PbSn1	1.23	4.9503	N.d.	100
S1-PbSn2	1.97	4.9491	N.d.	100
S1-PbSn3	2.85	4.9478	N.d.	100
S1-PbSn5	5.14	4.9468	N.d.	100
S1-PbSn5b	5.30	4.9464	3.30	96.70
S1-PbSn8	8.02	4.9467	3.73	96.27
S1-PbSn9	9.02	4.9466	5.66	94.34
S1-PbSn37	37.70	4.9468	32.51	67.48
S1-PbSn63	63.11	4.9470	55.35	44.65
S1-PbSn63_an	63.11	4.9439	48.85	42.47

**Table 5:** Pb lattice-parameter measurements of modern Cu–Sn–Pb ternary alloys

Sample name	Lattice parameter of Pb (Å) (e.s.d. = 8 × 10^−4^)	Sn content (at.%) from regression [equation (2)] ±0.50%
S2-CuSnPb5	4.9460	3.99
S2-CuSnPb10	4.9483	2.50
S2-CuSnPb16	4.9497	1.61

**Table 6:** Pb lattice-parameter measurements of historical bronze with the respective atomic percentage of Sn calculated from the calibrated relation (2)

	L-XRD		S-XRD	
Sample name	Pb lattice parameter (Å) (e.s.d. = 8 × 10^−4^)	Sn content (at.%) ±0.50%	Pb lattice parameter (Å) (e.s.d. = 10^−4^)	Sn content (at.%) ±0.50%
S3-Bronze39	4.9514	0.51	4.9504	0.26
S3-Bronze57	4.9532†	0†	4.9501	0.45
S3-Bronze98	4.9512	0.64	4.9502	0.38
S3-Bronze99	–	–	4.9528†	0†
S3-BronzeMg1	–	–	4.9502	0.38
S3-BronzeMg5	–	–	4.9502	0.38
S3-BronzeMgB2	–	–	4.9507	0.06
S3-BronzeMgB4	–	–	4.9500	0.51
S3-Pb	4.9522	0	4.9508	0
S3-PbSn50	4.9470	3.33	4.9463	2.88

† The Pb lattice parameter measured is slightly higher than that of pure Pb because a peak overlap with litharge (lead monoxide) is causing bias in the lattice-parameter determination.

## References

[bb1] Armstrong, J. T. (1991). *Electron Probe Quantitation*, edited by K. F. J. Heinrich & D. E. Newbury. Boston: Springer.

[bb2] Bernabale, M., Nigro, L., Montanari, D., Niveau-de-Villedary, A. M. & De Vito, C. (2019). *Mater. Charact.* **158**, 109957.

[bb3] Callister, W. D. Jr (1997). *Materials Science and Engineering: An Introduction*. New York: Wiley.

[bb4] Denton, A. R. & Ashcroft, N. W. (1991). *Phys. Rev. A*, **43**, 3161–3164. 10.1103/physreva.43.3161 9905387

[bb30] Dippel, A.-C., Liermann, H.-P., Delitz, J. T., Walter, P., Schulte-Schrepping, H., Seeck, O. H. & Franz, H. (2015). *J. Synchrotron Rad.* **22**, 675–687. 10.1107/S1600577515002222 PMC4416682 25931084

[bb5] Doebelin, N. & Kleeberg, R. (2015). *J. Appl. Cryst.* **48**, 1573–1580. 10.1107/S1600576715014685 PMC4603273 26500466

[bb6] Dreyer, W. & Müller, W. H. (2000). *Int. J. Solids Struct.* **37**, 3841–3871.

[bb7] Dungworth, D. B. (1997). *Internet Archaeol.* **2**, https://doi.org/10.11141/ia.2.2.

[bb8] Fecht, H. J. & Perepezko, J. H. (1989). *Metall. Trans. A*, **20**, 785–794.

[bb9] Harris, P. G., Chaggar, K. S. & Whitmore, M. A. (1991). *Soldering Surf. Mount Techn.* **7**, 20–23.

[bb10] Le Bail, A. (2005). *Powder Diffr.* **20**, 316–326.

[bb11] Magomedov, M. N. (2020). *Solid State Commun.* **322**, 114060.

[bb12] Nyyssönen, T., Ruusila, V., Kallio, M., Vuorinen, P., Valtonen, K. & Kuokkala, V.-T. (2012). *Finnish J. Tribology*, **31**, 5–11.

[bb13] Oberschmidt, J., Kim, K. K. & Gupta, D. (1982). *J. Appl. Phys.* **53**, 5672–5677.

[bb14] Peterson, V. K. (2005). *Powder Diffr.* **20**, 14–17.

[bb15] Rietveld, H. M. (1969). *J. Appl. Cryst.* **2**, 65–71.

[bb16] Ruusila, V., Nyyssönen, T., Kallio, M., Vuorinen, P., Lehtovaara, A., Valtonen, K. & Kuokkala, V.-T. (2013). *Proc. Inst. Mech. Eng. Part J J. Eng. Tribology*, **227**, 878–887.

[bb17] Scott, D. A. (2002). *Copper and Bronze in Art: Corrosion, Colorants, Conservation*. Los Angeles: Getty Conservation Institute.

[bb18] Scott, D. A., Podany, J. & Considine, B. B. (1994). *Ancient & Historic Metals: Conservation and Scientific Research, Proceedings of a Symposium Organized by the J. Paul Getty Museum and the Getty Conservation Institute, November 1991*. Marina del Rey: Getty Conservation Institute.

[bb19] Shilstein, S., Berner, A., Feldman, Y., Shalev, S. & Rosenberg, Yu. (2019). *STAR Sci. Tech. Archaeol. Res.* **5**, 29–35.

[bb20] Sidot, E., Kahn-Harari, A., Cesari, E. & Robbiola, L. (2005). *Mater. Sci. Eng. A*, **393**, 147–156.

[bb21] Straumanis, M. E. (1949). *J. Appl. Phys.* **20**, 726–734.

[bb22] Swanson, H. E. & Tatge, E. (1953). *Standard X-ray Diffraction Patterns*, US National Bureau of Standards Circular 539, Vol. 1, pp. 34–35. Washington, DC: US Government Printing Office.

[bb23] Thomas, S. P. (2007). *Casting Copper-Base Alloys*, 2nd ed. American Foundry Society.

[bb24] Tyzack, C. & Raynor, G. V. (1954). *Acta Cryst.* **7**, 505–510.

